# The Olive Leaves Extract Has Anti-Tumor Effects against Neuroblastoma through Inhibition of Cell Proliferation and Induction of Apoptosis

**DOI:** 10.3390/nu13072178

**Published:** 2021-06-24

**Authors:** Fabio Morandi, Veronica Bensa, Enzo Calarco, Fabio Pastorino, Patrizia Perri, Maria Valeria Corrias, Mirco Ponzoni, Chiara Brignole

**Affiliations:** 1 Stem Cell Laboratory and Cell Therapy Center, IRCCS Istituto Giannina Gaslini, 16147 Genova, Italy; fabiomorandi@gaslini.org; 2 Laboratory of Experimental Therapies in Oncology, IRCCS Istituto Giannina Gaslini, 16147 Genova, Italy; veronicabensa@gaslini.org (V.B.); enzocalarco@gaslini.org (E.C.); fabiopastorino@gaslini.org (F.P.); patriziaperri@gaslini.org (P.P.); mariavaleriacorrias@gaslini.org (M.V.C.); mircoponzoni@gaslini.org (M.P.)

**Keywords:** olive leaf extract, phytochemicals, neuroblastoma, anti-tumor effects, drugs combination

## Abstract

Neuroblastoma (NB) is the most common extra-cranial solid tumor of pediatric age. The prognosis for high-risk NB patients remains poor, and new treatment strategies are desirable. The olive leaf extract (OLE) is constituted by phenolic compounds, whose health beneficial effects were reported. Here, the anti-tumor effects of OLE were investigated in vitro on a panel of NB cell lines in terms of (i) reduction of cell viability; (ii) inhibition of cell proliferation through cell cycle arrest; (iii) induction of apoptosis; and (iv) inhibition of cell migration. Furthermore, cytotoxicity experiments, by combining OLE with the chemotherapeutic topotecan, were also performed. OLE reduced the cell viability of NB cells in a time- and dose-dependent manner in 2D and 3D models. NB cells exposed to OLE underwent inhibition of cell proliferation, which was characterized by an arrest of the cell cycle progression in G0/G1 phase and by the accumulation of cells in the sub-G0 phase, which is peculiar of apoptotic death. This was confirmed by a dose-dependent increase of Annexin V+ cells (peculiar of apoptosis) and upregulation of caspases 3 and 7 protein levels. Moreover, OLE inhibited the migration of NB cells. Finally, the anti-tumor efficacy of the chemotherapeutic topotecan, in terms of cell viability reduction, was greatly enhanced by its combination with OLE. In conclusion, OLE has anti-tumor activity against NB by inhibiting cell proliferation and migration and by inducing apoptosis.

## 1. Introduction

Neuroblastoma (NB), a neural crest-derived pediatric cancer, is the most common extra-cranial solid tumor of infancy, accounting for about 7% of all malignancy diagnosed under the age of 15 years [1,2]. It is the most common cancer diagnosed during the first year of life, with a median age at diagnosis of 17 months [2]. A wide clinical heterogeneity characterizes NB disease, ranging from cases of spontaneous regression to a highly metastatic disease already at diagnosis. Based on disease staging and risk factors assessment, the international NB risk group (INRG) stratifies patients into very low, low, intermediate, and high risk. This classification addresses the type of treatment to be undertaken [3]. To date, the prognosis for the high-risk NB-affected patients remains poor. Indeed, although the application of an intensive treatment schedule, which involves a phase of induction chemotherapy followed by surgery, myeloablative therapy combined with hematopoietic stem cells transplantation and local radiotherapy, and maintenance with anti-disialoganglioside (GD2) antibody plus isotrenoin, the survival rate for high-risk NB patients is less than 50% [3]. This scenario challenges the researchers to find out new therapies to support the standard of care treatment regimens, with the aim of improving the clinical outcome of these children.

Natural compounds represented for a long time the main source of therapeutic agents [4]. Historically, they have been widely employed for the prevention and cure of both physical and mental illness [5]. Furthermore, they have also been of pivotal importance in drug discovery [6]. It is noteworthy that many of the drugs approved by the Food and Drug Administration (FDA) and/or by the European Medical Agency (EMA) are based on plant derivatives [7]. The Vinca alkaloids, derived from the periwinkle plant Catharanthus roseus and the terpene Paclitaxel from Taxus baccata, are among the most successful plant-derived therapeutic agents approved for clinical use in oncology [8].

The concept of using natural derivatives, endowed with bioactive properties, as food supplement to maintain well-being, to ameliorate the health and lifestyle, to improve and sustain immunity responses, as well as to prevent and/or treat pathological conditions has again emerged in the last few years. As a consequence, the pharmaceutical industry invested enormous resources, economic and otherwise, leading to a booming industry branch [9].

Plant-derived bioactive molecules, also called phytochemicals, are widely distributed within fruits, seeds, vegetables, legumes, and leaves. The Mediterranean diet has been reported to contribute to well-being and has protective effects against neurodegenerative and cardiovascular diseases, as well as against cancer [10,11,12]. Specifically, the consumption of extra virgin olive oil (EVOO) in the diet leads to protective effects in the Mediterranean population. These beneficial properties can be ascribed to the high content of bioactive molecules, most of which are phenolic compounds (e.g., oleuropein, tyrosol, and hydroxytyrosol), within EVOO [13]. It is noteworthy that the olive leaf extract (OLE) is characterized by an even higher concentration of phenolic compounds with respect to EVOO [13]. Indeed, olive leaves are mainly constituted of secoiridoids (e.g., oleuropein, dimethyloleuropein), flavonoids (e.g., apigenin and luteolin), as well as other phenolics (e.g., hydroxytyrosol, tyrosol).

Phenolic compounds contained in OLE were demonstrated to be responsible for cell proliferation inhibition and apoptosis induction, in vitro, in different tumor models (i.e., breast cancer, pancreatic tumor, and leukemia) [13,14,15,16]. In addition, OLE improves the response of glioblastoma cells to standard treatments by modulating miRNA expression [17]. With this background, we here investigated the anti-tumor effects and relative mechanisms of action of OLE in monolayer (2D) and tridimensional (3D) models of NB in vitro. Moreover, the effects of combining OLE with topotecan, which is a chemotherapeutic used for the treatment of relapsed/refractory high-risk NB patients (Topotecan plus Temozolomide, TOTEM protocol, [18]), were also investigated.

## 2. Materials and Methods

### 2.1. Cell Lines

Human NB cell lines (HTLA-230, IMR-32, SH-SY5Y, and SK-N-AS [19,20,21,22]), HA-CAT cells (human keratinocytes [23]) and B-end (murine brain endothelial cells [24]) were cultured in Dulbecco’s Modified Eagle Medium (D-MEM) and RPMI-1640 supplemented with 10% of heat-inactivated fetal bovine serum (FBS), 50 IU/mL penicillin G, 50 μg/mL streptomycin sulphate, and 2 mM L-glutamine. Cells were periodically tested for mycoplasma contamination by polymerase chain reaction (PCR) assay, and characterized by cell proliferation and morphology evaluation, and also authenticated by multiplex short-tandem repeat profiling by BMR Genomics (Padova, Italy).

### 2.2. Olive Leaf Extract (OLE)

In this study, we used an aqueous Olive Leaf Extract (OLE; OLIVUM, EvergreenLife, San Giovanni al Natisone, Italy), which contains oleuropein, hydrohytyrosol, tyrosol, elenolic acid, and rutin, as assessed thanks to high performance liquid cromatography-mass spectrometry (HPLC-MS) by manufacturers. The mean concentration of each single element is reported in Table 1.

### 2.3. Cell Proliferation: Carboxyfluorescein Succinimidyl Ester (CFSE Assay)

NB cell lines were labeled with CFSE, following the manufacturer’s instruction (CellTrace^TM^ CFSE Cell Proliferation Kit, ThermoFisher Scientific, Waltham, MA, USA) and then seeded in 6-well plates (1.8−2.8 × 10^5^ cells/well, depending on the cell line used). The day after seeding, cells were treated with increasing doses (50–300 μM) of OLE, which were administered on the basis of oleuropein concentration within the extract. After 72 h (h) of treatment, cells were harvested and analyzed by flow cytometry (FCM).

### 2.4. Apoptosis: Annexin-V Assay

NB cell lines were seeded in 6-well plates (1.8−2.8 × 10^5^ cells/well, depending on the cell line used). The day after seeding, cells were treated with OLE (50–300 µM). After 72 h, cells were harvested and processed for apoptosis detection by the Annexin V-FITC kit (Beckman Coulter, Brea, CA, USA), following the manufacturer’s instructions. In some experiments, cells, before being treated with OLE, were pre-treated with the pan-caspases inhibitor Q-VD-OPh (Sigma-Aldrich, St. Louis, MO, USA) at 30 µM concentration for 30 min.

### 2.5. Citotoxicity

#### 2.5.1. Tetrazolium Salt (MTS) Cell Viability Assay in 2D Culture

NB cell lines and healthy control cells (HA-CAT and B-end) were seeded in 96-well plates (4−9.6 × 10^3^ cells/well, depending on the cell line used). The day after seeding, cells were treated with OLE, in quadruplicate for each experimental condition, as already detailed above. After 48–120 h, cells were processed to determine the cytotoxic effects mediated by OLE treatment. The MTS tetrazolium compound assay was used, according to the manufacturer’s instructions (CellTiter 96^®^ Aqueous One Solution Cell Proliferation Assay, Promega Italia, Milano, Italy). The MTS tetrazolium compound is converted by metabolically active cells to produce a colored formazan, whose amount is proportional to the number of viable cells. In some experiments, NB cell lines and healthy control cells were treated with OLE for 18 h (namely “short-treatment” experiments). Then, OLE was removed, and cells were recovered with fresh complete medium for an additional 54 and 102 h. At the end point time, 72 and 120 h respectively, the MTS assay was used.

In a separate set of experiments, NB cells seeded as described above were treated with a combination of OLE and topotecan (TOPO, Sigma-Aldrich, St. Louis, MO, USA). The doses of TOPO were chosen on the basis of the literature [25]. After 96 h of treatment, cells were subjected to the MTS assay to determine cytotoxic effects. Then, the results were analyzed by the use of the Compusyn online software (available at http://www.combosyn.com (accessed on 10 June 2021)/, Combosyn, Inc., Paramus, NJ, USA), with the aim to assess synergism, addiction, and/or antagonism between OLE and TOPO.

#### 2.5.2. CellTiter-Glo^®^ Assay in 3D Culture

The NB cell lines IMR-32 and SH-SY5Y (2 × 10^3^ and 1 × 10^3^, respectively) were seeded in ultra-low-attachment 96-well plates (Corning, Steuben, NY, USA). After a three-day culture, when a single spheroid per well was formed, cells were treated continuously in quadruplicate for each experimental condition, with OLE as already detailed above. After 72 and 120 h, spheroids were transferred into black-walled 96-well plates and assayed for cell viability determination through the CellTiter-Glo^®^ 3D Cell Viability Assay (Promega, Italia, Milano, Italy).

Tumor spheroids were also used to assess the potential combination of OLE and TOPO in terms of cell viability reduction. The combination of OLE and TOPO, leading to the best result in the 2D model (OLE 200 μM + TOPO 10 nM), was used as a proof of concept assay in the 3D model. As already described above, treatment was administered continuously when a single spheroid per well was formed. After 96 h, the cell viability of each tumor spheroid was determined.

### 2.6. Cell Cycle Analysis

NB cell lines (IMR-32 and HTLA-230) were seeded in 6-well plates (2.8 × 10^5^ and 3 × 10^5^ cells/well, respectively). The day after seeding, cells were treated with 200 and 300 µM of OLE. Then, 72 h later, cells were harvested and labeled with Nuclear Green CCS1 (abcam, Cambridge, United Kingdom) to monitor the cell cycle progression in live cells. According to the manufacturer’s instructions, cells were incubated with Nuclear Green for 1 h at 37 °C. Then, cell cycle progression was assessed by FCM.

### 2.7. Signaling

NB cell lines were seeded in 6-well plates, as already mentioned above. The day after seeding, cells were treated with 200 and 300 μM of OLE, as already described above. After 72 h of treatment, cells were collected and processed for cell signaling evaluation by FCM. Specifically, the expression of cleaved caspase 3, cleaved caspase 7, cleaved caspase 8, p53, cyclin D1, Bcl-2, phospho-Bcl-2, NF-kB p65, and phospho-NF-kB p65 was determined, according to the manufacturer’s instruction (Cell Signaling Technology Inc., Danvers, MA, USA).

### 2.8. Reactive Oxigen Species (ROS) Production

For the detection of total ROS, NB cell lines (IMR-32 and HTLA-230) were labeled with H2DCFDA (ThermoFisher Scientific, Waltham, MA, USA) according to previously published protocols [26], and seeded in clear-bottom 96-well black plates (2 × 10^4^ cells/well). H2DCFDA is a cell permeant non-fluorescent dye which in the presence of ROS is oxidized, becoming green fluorescent. The day after seeding, cells were treated with increasing concentrations of OLE. After 24 h of exposure to OLE, plates were centrifuged (1400 rpm for 7 min). Then, cells were washed and recovered with phosphate buffered saline (PBS). ROS production was determined by the measurement of green fluorescence (485 nm/535 nm, excitation/emission).

### 2.9. Migration: Scratch Test

The NB cell lines IMR-32 and SH-SY5Y (8 × 10^5^ and 4.5 × 10^5^, respectively) were seeded in 6-well plates. Two days after seeding, when cells were almost confluent, a vertical wound was made through the cell monolayer, using a 200 µL pipette tip. Then, cells were treated with OLE (200 and 300 µM). At the time of treatment (T0) and at 4, 24, 36, and 48 h, cells were photographed, and the width of the scratch was determined and recorded by the use of ImageJ software (Rasband, W.S.; ImageJ, US. National Institutes of Health, Bethesda, MD, USA, http://imagej.nih.gov/ij/, 1997–2012; accessed on: 2019–2020).

### 2.10. Statistics

All the experiments were performed at least three times with similar results. Each experimental condition, for the assays performed in 96-well plates, was carried out in quadruplicate.

Differential findings among the experimental groups were determined by one-way analysis of variance, with Tukey’s multiple comparison test, using GraphPad Prism 5 (GraphPad Software v5.0, San Diego, CA, USA). Slope differences of the migration assay were determined by the use of GraphPad Prism 5.

## 3. Results

### 3.1. Olive Leaf Extract (OLE) Has Cytotoxic Effects on a Panel of NB Cell Lines

**Figure 1:**
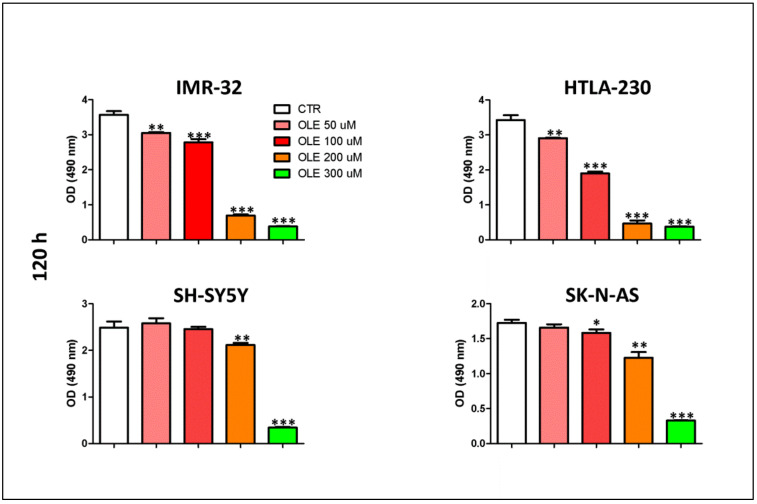
Dose-dependent effects of OLE treatment on the cell viability of NB cells. Data represent the results of MTS assays performed after 120 h of OLE treatment. Optical density (OD, at 490 nm) was recorded by the use of the TECAN micro-plate reader, Infinite 200 (Tecan Life Sciences). Data are expressed as mean ± SD of three independent experiments (* *p* < 0.05, ** *p* < 0.01, *** *p* < 0.001 vs CTR). CTR: control; OLE: olive leaf extract; uM: micromolar; nm: nanometer; h: hours.

The cytotoxic effects of OLE were evaluated in vitro on a panel of human NB cell lines. Figure 1 shows that all NB cells analyzed are sensitive to OLE treatment, at a different extent and in a dose-dependent manner. Specifically, the cell viability of IMR-32 and HTLA-230 cells was reduced by the lowest dose of OLE, while only the highest concentrations of OLE significantly affected the cell viability of SH-SY5Y and SK-N-AS.

**Figure 2:**
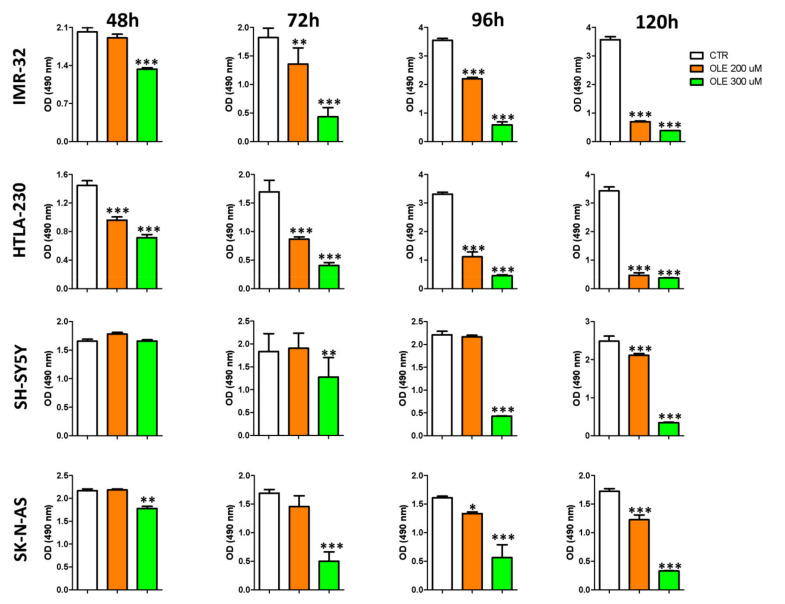
Time-dependent effects of OLE treatment on the cell viability of NB cells. The MTS assay was performed after 48, 72, 96, and 120 h of OLE treatment. Optical density (OD, at 490 nm) was recorded by the use of the TECAN micro-plate reader, Infinite 200 (Tecan Life Sciences). Data are expressed as mean ± SD of three independent experiments (* *p* < 0.05, ** *p* < 0.01, *** *p* < 0.001 vs. CTR). CTR: control; OLE: olive leaf extract; uM: micromolar; h: hours; nm: nanometer.

A time-course experiment, focused only on the two mainly effective doses (200 and 300 μM), was further performed. Figure 2 shows that OLE affected the cell viability of NB cells also in a time-dependent way. IMR-32 and HTLA-230 cell lines were, as expected, more sensitive to OLE treatment compared to SH-SY5Y and SK-N-AS, whose cell viability was reduced only after a longer time of exposure to OLE.

Experiments performed on healthy cell lines (B-end and HA-CAT) revealed that only the 300 μM dose of OLE resulted in a moderate reduction of cell viability in one out of two control cell lines tested after a long exposure time (120 h; Supplementary Figure S1).

Accounting for the potential limitation of the in vivo use of OLE at μM concentrations in managing NB disease, short-treatment experiments were performed. As illustrated in Supplementary Figure S2A,B, the short-treatment exposure was almost ineffective against healthy control cells, while NB cells exposed for 18 h to OLE underwent a significant dose-dependent reduction of cell viability at both the endpoints analyzed.

**Figure 3:**
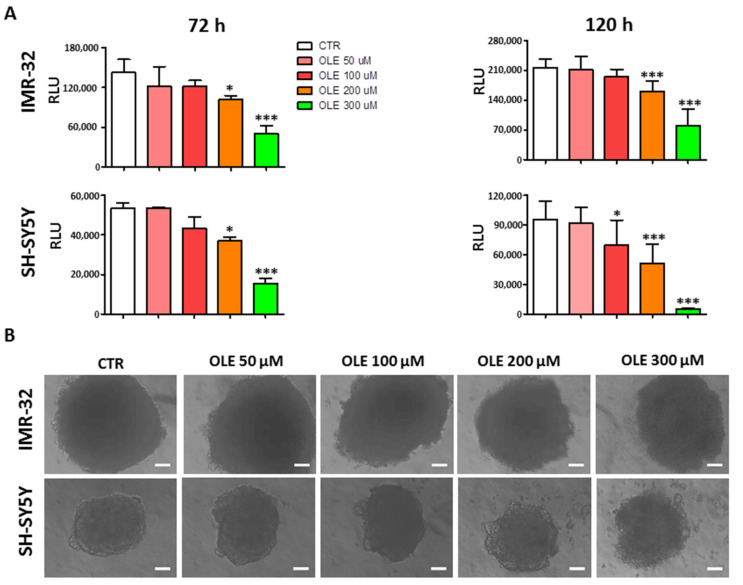
Dose- and time-dependent effects of OLE treatment on the viability of NB spheroids. (**A**) Cell viability determined at indicated time points, thanks to the CellTiter-Glo^®^ 3D Cell Viability assay. Luminescence, produced only by metabolically active cells, was recorded by TECAN Infinite 200, and recorded as relative luminescence unit (RLU). Data are expressed as mean ± SD of three independent experiments. * *p* < 0.05, *** *p* < 0.001 vs. CTR). (**B**) Representative phase-contrast microscopy images of spheroids taken at 120 h post OLE treatment. The ImageJ processing program was used. Scale bar: 100 μm. CTR: control; OLE: olive leaf extract; uM: micromolar; h: hours.

Experiments were also performed in a 3D culture system, which resembles the peculiar features of solid tumors and better reflects and predicts the response to a given therapy. Figure 3 shows the results of cytotoxic experiments carried out on IMR-32 and SH-SY5Y tumor spheroids. OLE maintained its ability to significantly reduce the cell viability of NB spheroids in a dose- and time-dependent manner at the two mainly effective doses (200 and 300 μM).

### 3.2. OLE Inhibits Cell Proliferation of NB Cell Lines through a G0/G1 Cell Cycle Arrest

**Figure 4:**
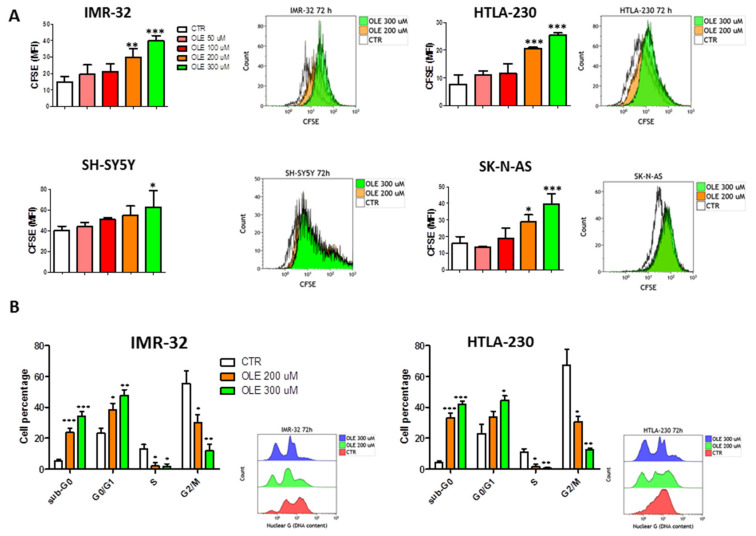
Inhibition of cell proliferation, through a G0/G1 cell cycle arrest, mediated by OLE treatment. (**A**) The green fluorescence intensity of Carboxyfluorescein Succinimidyl Ester (CFSE) was recorded by FCM. According to the dilution of the dye, more fluorescence means less proliferation. Insets show representative FCM experiments of CFSE assay. (**B**) Cell cycle progression of IMR-32 and HTLA-230 determined by Nuclear Green labeling. Insets show representative cell cycle progression assay, analyzed by FCM. Data are expressed as mean ± SD of three independent experiments (* *p* < 0.05, ** *p* < 0.01, *** *p* < 0.001 vs. CTR). CTR: control; OLE: olive leaf extract; uM: micromolar; MFI: mean fluorescence intensity.

NB cell lines treated with increasing doses of OLE for 72 h underwent a dose-dependent reduction of cell proliferation, as assessed by the CFSE assay (Figure 4A). Again, such effect was achieved mainly with 200 and 300 μM of OLE and was more evident in IMR-32 and HTLA-230 than in SH-SY5Y and SK-N-AS cells. Thus, cell cycle progression was further investigated in the two former cell lines. Both IMR-32 and HTLA-230 cells went to a G0/G1 cell cycle arrest after 72 h of treatment with 200 and 300 μM of OLE (Figure 4B). This was accompanied by the almost complete disappearance of the S phase and the progressive reduction of the G2/M phase in both the cell lines analyzed. Moreover, as a result of G0/G1 cell cycle arrest, OLE treatment determined a significant increase of cells in the sub-G0 phase (Figure 4B). Furthermore, the G0/G1 arrest correlated with a dose-dependent upregulation of wild-type p53 and cyclin-D1 protein expression (Supplementary Figure S3A,B).

### 3.3. OLE Induces Apoptosis of NB Cells

**Figure 5:**
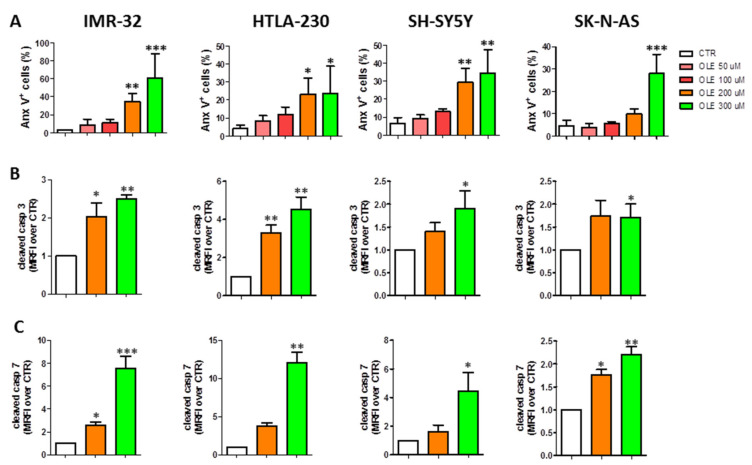
Dose-dependent induction of NB cells apoptosis after OLE treatment. (**A**) Anx-V+ cells determined by FCM. Protein expression levels of the executioner caspases 3 (**B**) and 7 (**C**) assessed by FCM. Data are expressed as mean ± SD of three independent experiments (* *p* < 0.05, ** *p* < 0.01, *** *p* < 0.001 vs. CTR). OLE: olive leaf extract; CTR: control; uM: micromolar; MRFI: mean ratio fluorescence intensity.

Additional experiments were performed to assess whether the increasing percentage of sub-G0 cells (namely apoptotic) after OLE exposure was related to the induction of apoptosis in NB cells. As shown in Figure 5A and Supplementary Figure S4A, OLE-treated cells underwent a dose-dependent apoptosis, as underlined by the increased percentage of Anx-V+ cells, which was significant, compared to control cells, at the highest doses of OLE used. Moreover, in all the NB cell lines analyzed, OLE treatment led to a dose-dependent activation of the executioner caspases 3 and 7 (Figure 5B,C and Supplementary Figure S4B,C). Finally, the apoptotic cell death induced by OLE treatment was significantly rescued by the pre-treatment of the cells with the pan-caspases inhibitor Q-VD-OPh, further supporting the results obtained (Supplementary Figure S5A,B). Experiments were performed to assess the signaling pathways involved in the apoptotic cell death. The significant increase of cleaved caspase 8 protein expression levels clearly indicates the involvement of the extrinsic pathway (Supplementary Figure S6A,B). On the other hand, the significant upregulation of phospho-Bcl-2, only at the highest dose of OLE (300 µM) used, seems to suggest also the contemporary activation of the intrinsic apoptotic pathway (Supplementary Figure S6A,B). This last pathway could be implicated as a consequence of the increased production of total ROS in response to OLE treatment (Supplementary Figure S7).

### 3.4. OLE Activates the NF-kB Pathway

In order to investigate whether OLE treatment may be involved in inflammatory pathways, we also investigated the protein expression levels of NF-kB and phospho-NF-kB. As depicted in Supplementary Figure S8, OLE treatment led to an increased expression of phospho-NF-kB, while the native form of NF-kB remained unaltered.

### 3.5. OLE Inhibits Migration of IMR-32 and SH-SY5Y Cells

**Figure 6:**
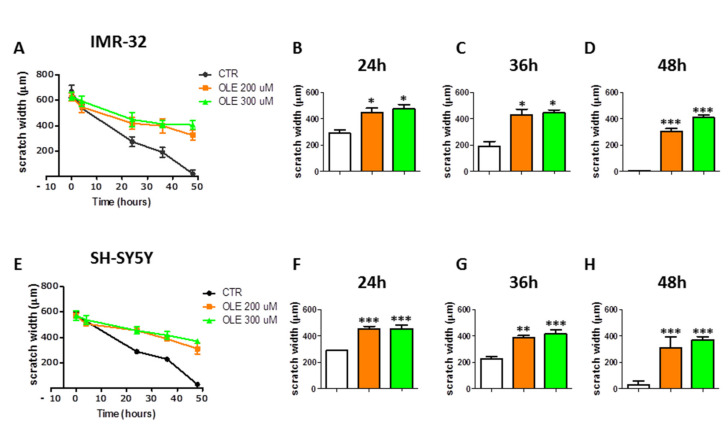
Effects of OLE on the migration of NB cells. (**A**) and (**E**) represent the wound closure curves of CTR vs. OLE-treated cells of IMR-32 and SH-SY5Y, respectively. (**B**–**D**) and (**F**–**H**) show the differences of width wound among experimental groups at the indicated time points. Data are expressed as mean ± SD of three independent experiments (* *p* < 0.05, ** *p* < 0.01, *** *p* < 0.001 vs. CTR). CTR: control; OLE: olive leaf extract; uM: micromolar; µm: micrometer; h: hours.

The scratch assay, performed on IMR-32 and SH-SY5Y NB cells, demonstrated that OLE treatment inhibits the migration of NB cells. Indeed, following OLE exposure, the IMR-32 and SH-SY5Y cells failed to completely heal the wound created in the cell monolayer, with respect to control untreated cells, over a period of 48 h (Figure 6). Applying the linear regression analysis, to compare wound closure curves, it resulted that the slopes of OLE-treated cells (either at 200 or 300 μM) were significantly different with respect to those of control untreated cells (*p* < 0.0001, for either IMR-32 or SH-SY5Y). The wound width of OLE-treated cells was significantly wider than in untreated cells at 24, 36, and 48 h time points (Figure 6 and Supplementary Figure S9).

### 3.6. OLE Synergizes with Topotecan against NB Cells

**Figure 7:**
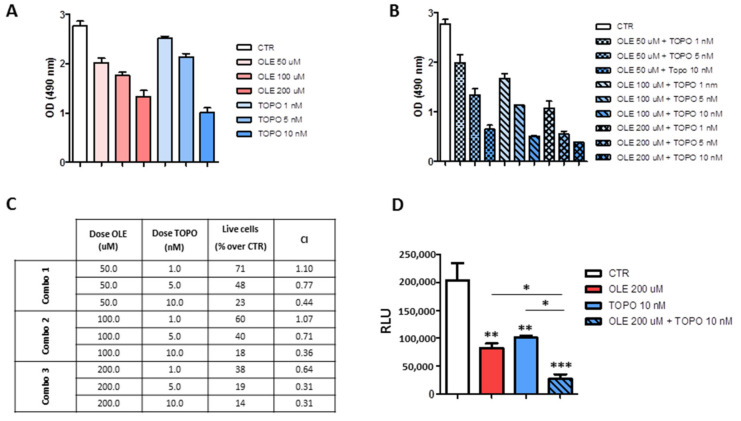
Effects of OLE and topotecan combination on the cell viability of IMR-32 and SH-SY5Y cells. (**A**) MTS assay showing the effects of the single drugs. (**B**) MTS assay showing the effects of drugs combination. (**C**) Table showing the combination index (CI) values for each OLE-TOPO combination. Combo 1 = OLE 50 μM plus TOPO 1, 5, and 10 nM; Combo2 = OLE 100 μM plus TOPO 1, 5, and 10; Combo 3 = OLE 200 μM plus TOPO 1, 5, and 10. CI < 1 means synergism. CI > 1 means antagonism. CI = 1 means addiction. (**D**) Viability assay showing the effects of OLE and TOPO, as single drugs and in combination, on tumor spheroids of IMR-32 cell line. Data are expressed as mean ± SD of three independent experiments (* *p* < 0.05, ** *p* < 0.01, *** *p* < 0.001). CTR: control; OLE: olive leaf extract; TOPO: topotecan; uM: micromolar; nM: nanomolar; OD: optical density; RLU: relative luminescence unit.

Proof-of-concept experiments of drug combination were performed with the aim to determine whether OLE could support conventional chemotherapy in the treatment of high-risk NB patients at relapse. Increasing doses of OLE (50, 100, 200 μM) and topotecan (TOPO, 1, 5, 10 nM [25]) were combined with each other. Figure 7A,B shows the results obtained by using “drugs” as single agents or in combination, respectively. The effects of drugs combination were determined thanks to the theorem of Chou and Talalay [27]. The combination index (CI) values allow distinguishing among synergism (CI < 1), addiction (CI = 1), and antagonism (CI > 1). As shown in Figure 7C, the combined administration of OLE and TOPO to IMR-32 cells, over 96 h of treatment, led to generalized synergistic effects except for two points with a CI > 1. More in detail, most of the OLE-TOPO combination analyzed produced either synergism (range of CI = 0.3–0.7) or moderate synergism (range of CI = 0.7–0.85). Furthermore, the two combinations (OLE 50 μM + TOPO 1 nM, CI = 1.1 and OLE 100 μM + TOPO 1 nM, CI = 1.07) with a CI > 1 produced a nearly addictive effect, in which the CI value was between 0.9 and 1.1 [27]. The best combination producing synergistic results (OLE 200 μM + TOPO 10 nm, CI = 0.31) was also tested against spheroids of the IMR-32 cell line. This combination significantly reduced the cell viability of tumor spheroids compared to every single drug used alone, further confirming the results obtained in monolayer-based assays (Figure 7D).

## 4. Discussion

To our knowledge, this study demonstrates for the first time the anti-tumor effects of olive leaf extract (OLE) against NB cells by investigating cell viability, cell proliferation, cell cycle progression, apoptosis, and migration in vitro. To date, only one report examined the anti-tumor efficacy of the single bioactive molecule oleuropein contained in OLE against the SH-SY5Y neuroblastoma cell line [28], but nothing is reported about the whole extract.

OLE has indeed the peculiarity to be constituted by a wide variety of phenolic compounds, whose health beneficial effects have been reported [29,30]. Several studies focused on the main constituents of OLE (i.e., oleuropein, hydroxytyrosol) as single molecules and demonstrated their anti-tumor properties in different tumor models [13]. Oleuropein, the most abundant phenolic compound present in OLE, was demonstrated to be effective in inducing apoptosis through cell cycle arrest and the activation of a mitochondrial signaling cascade in HeLa cells [31]. Moreover, oleuropein induced apoptosis and inhibited the cell proliferation of HepG2 human hepatoma cell line [32]. More recently, the pro-apoptotic properties of oleuropein were also confirmed by Antognelli et al., who demonstrated that oleuropein induces apoptosis in a non-small cell lung cancer cell line (A549) through the upregulation of mitochondrial glyoxalase 2 [33]. Anti-tumor effects were also reported for hydroxytyrosol (HT), who reduced the proliferation of human colon adenocarcinoma cells through the inhibition of extracellular signal-regulated kinases (ERK) 1/2 and cyclin D1 [34]. Moreover, HT was also effective against prostate cancer cells through the induction of apoptosis and cell cycle arrest [35]. Luteolin and apigenin, although less concentrated, also demonstrated their potential as anti-tumor agents [36,37].

Neuroblastoma (NB) is most common solid tumor of pediatric age. The cure of high-risk NB patients is still challenging, and the possibility to find food supplements endowed with anti-tumor properties and to be used as adjuvant in combination therapies appears interesting. The whole olive leaf extract deserves a great amount of attention, because due to the cooperation of different bioactive molecules, it could have anti-tumor properties even more pronounced than every compound taken as a single agent.

Here, OLE administered on the basis of oleuropein concentration led to a time-and dose-dependent reduction of cell viability on a panel of human NB cell lines. The variable sensitivity of NB cells to OLE reflected the broad heterogeneity of NB tumors [38]. From a translational point of view, the use of µM concentration of OLE raises the question on the possible toxicity on normal tissues. At present, the knowledge on the safety profile of OLE in humans is still limited, even if it is reported that it is generally reliable and not toxic also at high doses [39,40]. Here, it was demonstrated that OLE is not toxic against healthy control cells, as assessed by comparing long and continuous exposure to OLE vs. short-term treatment. These results are encouraging, and although deepened safety and toxicology studies are needed for future translational clinical applications, it seems that the use of OLE as adjuvant could be beneficial while not toxic by using an appropriate therapeutic window.

Monolayer culture systems (2D) have been for decades the best models for drugs testing; nevertheless, the results obtained were frequently inconsistent when moving to the in vivo models. Tumor spheroids better mimic the architecture features of solid tumors compared to 2D. They are characterized by a complex structure that recapitulates the complexity of solid tumors in terms of cell-to-cell interaction, cell-to-extracellular matrix interconnection, differential access to oxygen and to nutrient by the cell layers of the structure, and the differential growth rate of the layers [41]. All these features, together with their potential to better reflect and predict the response to therapy, led tumor spheroids to emerge as a proper model for anti-cancer drug screening [42]. Cytotoxic experiments, performed on reliable and reproducible tumor spheroids, demonstrated that OLE reduced tumor cell viability in a time- and dose-dependent manner, confirming and validating the results obtained in 2D cultures. These translational results pave the way to future experiments in more complex and dynamic 3D settings and in in vivo pre-clinical models.

According to a previous study performed in melanoma [43], our work demonstrates that OLE is able to inhibit the proliferation of NB cells by arresting the cell cycle in the G0/G1 phase. In contrast, it was reported that oleuropein inhibited the cell proliferation of human hepatoma cells and colon cancer cells through a G2/M phase cell cycle arrest [44,45]. This finding underlies that OLE, due to the cooperation of different bioactive molecules, can act through different mechanisms with respect to the single molecules. Our results also demonstrate that NB cells exposed to OLE undergo a dose-dependent apoptotic cell death, which correlated with increased expression of the executioner caspases 3 and 7, in partial agreement with previous studies performed on melanoma [43].

The investigations performed to unravel the main signaling pathways activated in response to OLE treatment clearly indicate the activation of the extrinsic apoptotic pathway, which is underlined by the increased expression of the cleaved form of caspase 8. It is noteworthy that our data also demonstrate the increased expression of phospho-Bcl-2 at the highest dose of OLE. This result opens to several hypotheses. Indeed, the phosphorylation of Bcl-2 may be a consequence of the production of ROS in response to OLE treatment, finally leading to activate also the intrinsic apoptotic pathway [46]. On the other hand, the phosphorylation of Bcl-2 could represent a mechanism put in place by the cells to counteract oxidative stress-related DNA damage, as previously reported [47]. Indeed, total ROS, but not mitochondrial ROS, were increased by OLE treatment (data not shown).

Moreover, treatment of NB cells with OLE led to the activation of the NF-kB pathway, which is involved, among others, also in inflammation signaling [48,49]. The increased phosphorylation of NF-kB upon treatment with OLE could suggest the activation of a pro-inflammatory cascade. Such speculation needs further in vivo investigations to be confirmed. One of the key features of an anti-cancer bioactive molecule is also to interfere with the process of migration of cancer cells, which is responsible for cancer progression and metastatization. In previous studies, oleuropein as well as hydroxytyrosol were reported to be able to inhibit the migration of different cancer cell types [28,50,51]. In accordance, we here demonstrate that OLE is able to significantly inhibit the migration of NB cells, further substantiating its anti-tumor effectiveness.

Our study, performed on a panel of human NB cell lines, mostly agrees with the previous findings by Secme et al. [27], who demonstrated the anti-tumor effects of oleuropein against the SH-SY5Y NB cell line. From the results obtained, it is not possible to indicate whether OLE was advantageous in terms of anti-tumor effects compared to the single agent oleuropein. However, we can underline that OLE was demonstrated to be effective also in a short-time schedule of administration. Furthermore, the aqueous extract presents several advantages compared to oleuropein for future clinical application. Indeed, from a clinical point of view, the aqueous extract could be easily administered per os, which is feature particularly important for an adjuvant “drug” to be used in pediatric oncology. By contrast, oleuropein solubility could necessitate the development of specific pharmacological formulations to render it easily administrable [47].

The final aim of this study was to investigate the potential cooperation of OLE and the chemotherapeutic topotecan, which is used in the clinical practice to treat relapsed/refractory NB-affected patients. The possibility of combining chemotherapeutics with phytochemicals, which could increase their therapeutic index while having a safe toxic profile, sounds interesting and promising, especially for relapsed/refractory NB patients. The ability of OLE to increase the response of tumor cells to standard treatments has been already demonstrated in glioblastoma [17] and melanoma [43]. We here show that the combination of OLE and topotecan led to encouraging anti-tumor effects in a monolayer assay, with combination index values ranging from synergy to addiction. These results were further confirmed and supported by proof-of-concept experiments performed against NB tumor spheroids, where the combination of OLE and TOPO significantly reduced the cell viability of tumor spheroids compared to every single drug used alone.

## 5. Conclusions

In conclusion, the in vitro results here shown open up to deepened pre-clinical investigations before considering the possibility to translate OLE administration to the clinical setting, and to likely improve, in combination to standard treatments, the clinical outcome of high-risk NB patients.

## Figures and Tables

**Table 1:** Mean concentration of phytochemicals contained in OLE.

Compound	Mean Concentration
Oleuropein	2656 mg/L
Hydrohytyrosol	213 mg/L
Tyrosol	174 mg/L
Elenolic Acid	1393 mg/L
Rutin	237 mg/L

## Data Availability

Not applicable.

## Supplementary Materials

The following are available online at https://www.mdpi.com/article/10.3390/nu13072178/s1, Figure S1: Effects of long-term exposure to OLE treatment on the cell viability of healthy control; Figure S2: Effects of short-treatment exposure to OLE on the cell viability of NB cells and healthy controls; Figure S3: Upregulation of p-53 and cyclin-D1 protein expression levels after treatment with OLE; Figure S4: Apoptosis induction and upregulation of caspases 3 and 7 expression after OLE treatment. Figure S5: Rescue of the apoptotic cell death induced by OLE by pre-treatment with the pan caspases inhibitor Q-VD-OPh. Figure S6: Upregulation of cleaved caspase 8 and phospho-Bcl-2 after OLE treatment. Figure S7: Increased production of total ROS after OLE treatment. Figure S8: Upregulation of phospho-NF-kB after OLE treatment. Figure S9: Inhibition of migration of IMR-32 and SH-SY5Y cell lines after OLE treatment.
