# Supplementary material for: The Olive Leaves Extract Has Anti-Tumor Effects against Neuroblastoma through Inhibition of Cell Proliferation and Induction of Apoptosis
Source: Nutrients. 2021 Jun 24;13(7):2178. doi: 10.3390/nu13072178 (PMC8308225; doi:10.3390/nu13072178)
Supplement: Supplementary file 1 [file nutrients-13-02178-s001.zip › nutrients-1275769-supplementary.pdf]

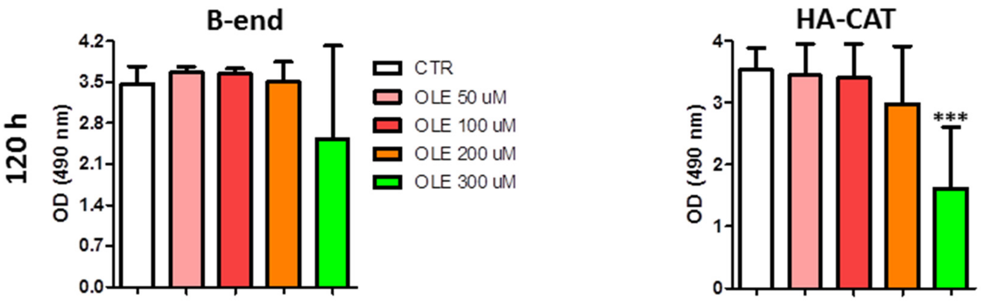

Supplementary Figure S1. Effects of long-term exposure to OLE treatment on the cell viability of healthy control cells. Data show the results of MTS assay carried out after 120 h of treatment with OLE. Optical density (OD, at 490 nm) was recorded by the use of the TECAN micro-plate reader, Infinite 200 (Tecan Life Sciences). Data are expressed as mean ± SD (*** $p < 0.001$ vs CTR). CTR: control; OLE: olive leaf extract; uM: micromolar; nm: nanometer; h: hours.

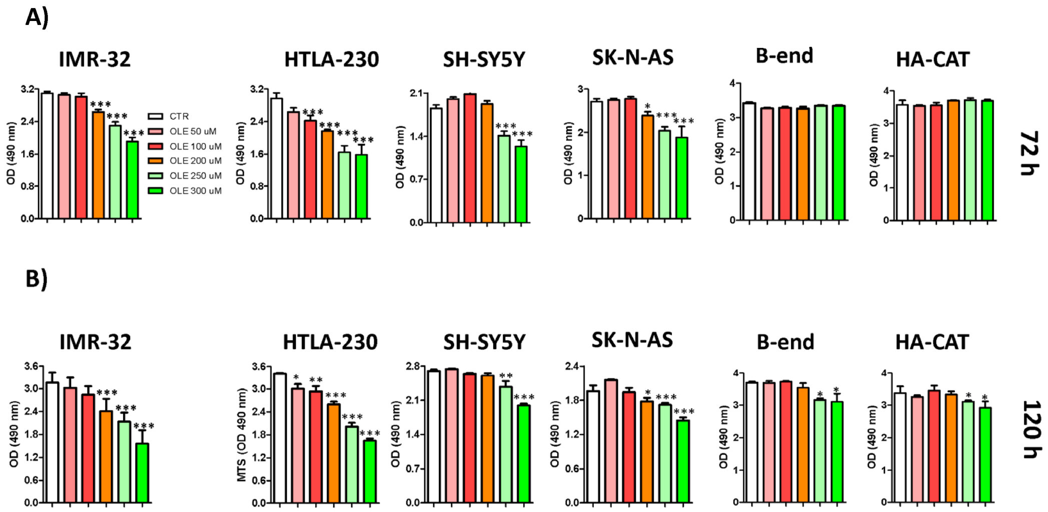

Supplementary Figure S2. Effects of short-treatment exposure to OLE on the cell viability of NB cells and healthy controls. (**A**) and (**B**) show the results of MTS assay carried out at 72 and 120 h, respectively. Optical density (OD, at 490 nm) was recorded by the use of the TECAN micro-plate reader, Infinite 200 (Tecan Life Sciences). Data are expressed as mean ± SD (* $p < 0.05$, ** $p < 0.01$, *** $p < 0.001$ vs CTR). CTR: control; OLE: olive leaf extract; uM: micromolar; nm: nanometer; h: hours.

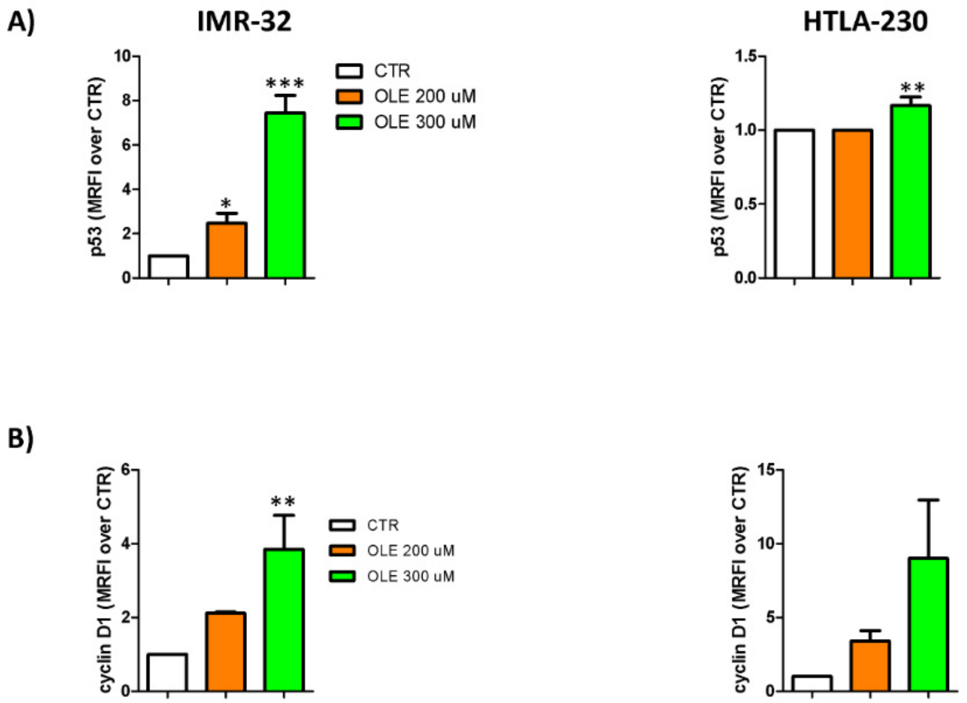

Supplementary Figure S3. Up-regulation of p-53 and cyclin-D1 protein expression levels after treatment with OLE. Data represent the protein expression levels of (**A**) p-53 and (**B**) cyclin-D1, determined by FCM as mean ratio fluorescence intensity (MRFI) over control levels. Data are expressed as mean ± SD (* $p < 0.05$, ** $p < 0.01$, *** $p < 0.001$*vs* CTR).

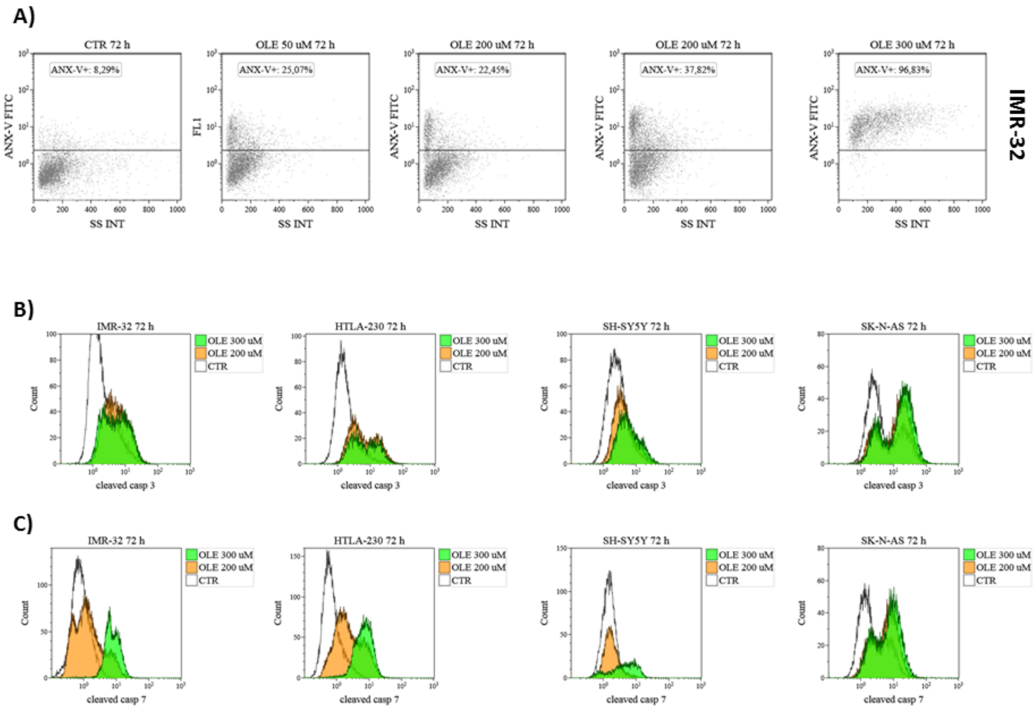

Supplementary Figure S4. Apoptosis induction and upregulation of caspases 3 and 7 expression after OLE treatment. Representative flow citometry (FCM) analyses of (**A**) Annexin-V (ANX-V) binding assay, (**B**) cleaved caspase 3 and (**C**) cleaved caspase 7 expression.

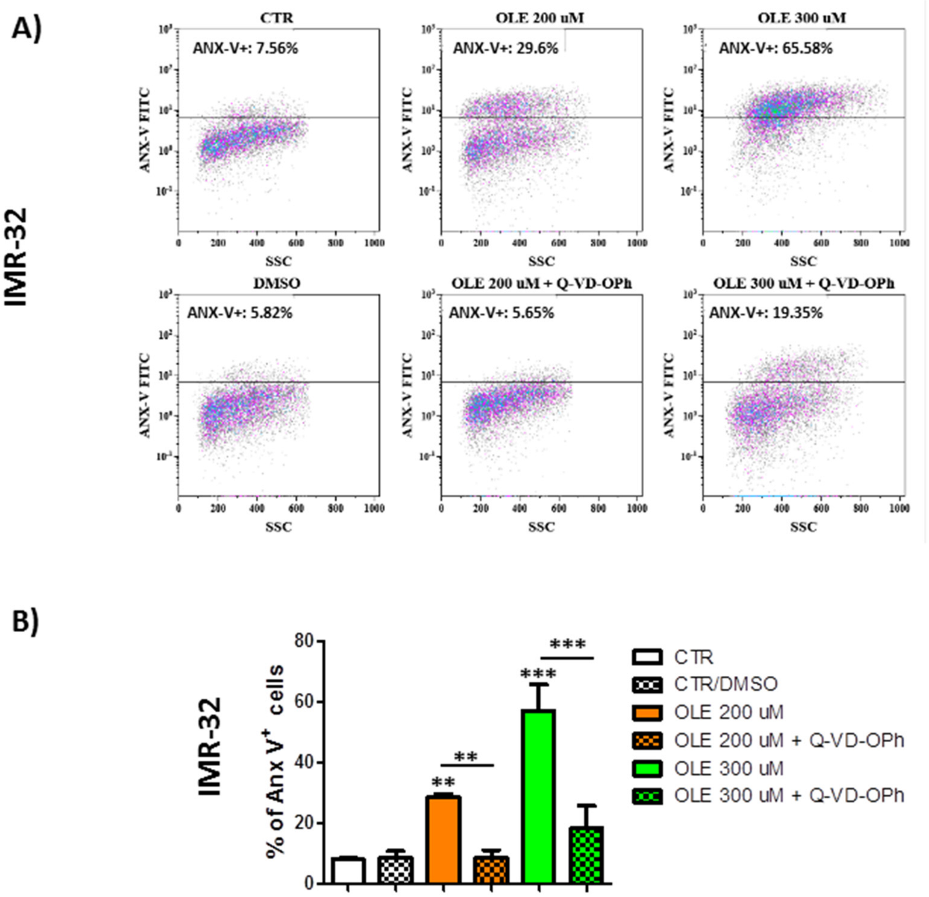

Supplementary Figure S5. Rescue of the apoptotic cell death induced by OLE by pre-treatment with the pan caspases inhibitor Q-VD-OPh. (**A**) Representative FCM analyses of ANX-V binding assay. (**B**) Data show the results obtained by pre-treating NB cells with the pan-caspases inhibitor Q-VD-OPh. Data are expressed as ± SD (** $p < 0.01$ OLE 200 uM vs CTR, and OLE 200 uM vs OLE 200 uM vs OLE 200 uM + Q-VD-OPh; *** $p < 0.001$OLE 300 uM vs CTR and OLE 300 uM vs OLE 300 uM vs OLE 300 uM vs OLE 300 uM + Q-VD-OPh).

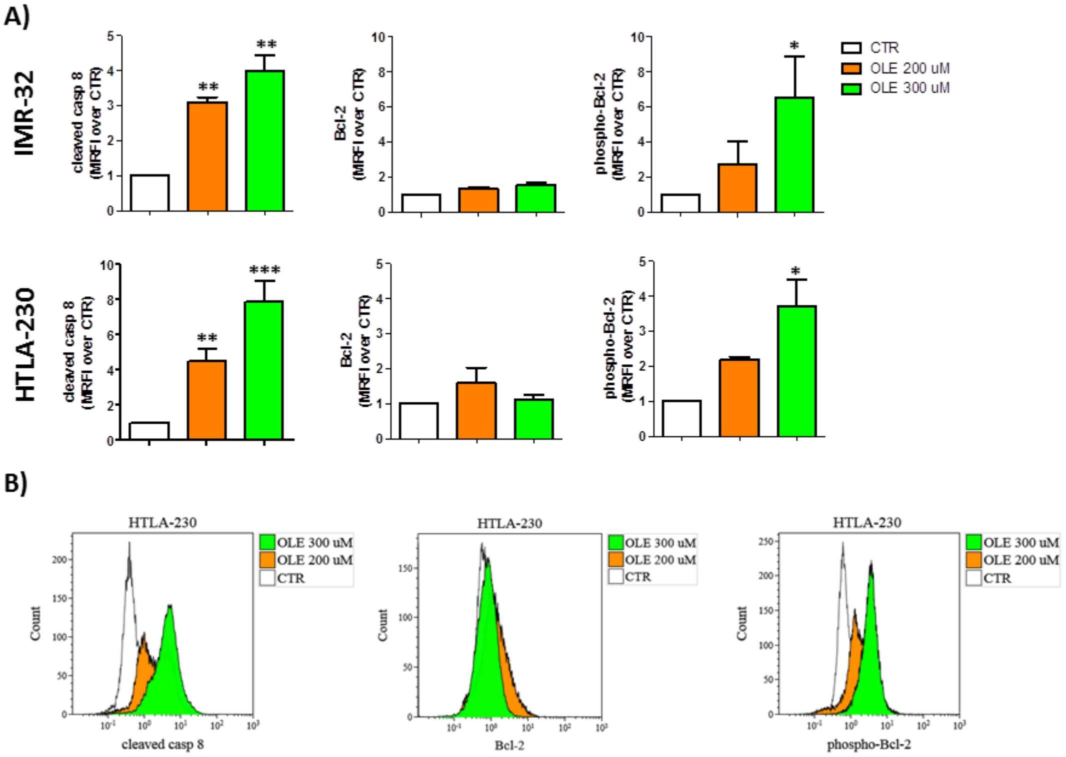

Supplementary Figure S6. Up-regulation of cleaved caspase 8 and phospho-Bcl-2 after OLE treatment. (**A**) Data show the results of cleaved caspase 8, Bcl-2 and phospho-Bcl-2 protein expression levels determined by FCM as MRFI over CTR. (**B**) Representative FCM analyses of cleaved caspase 8, Bcl-2 and phospho-Bcl-2 protein expression levels. Data are expressed as mean ± SD (* $p < 0.05$, ** $p < 0.01$, *** $p < 0.001$ vs CTR).

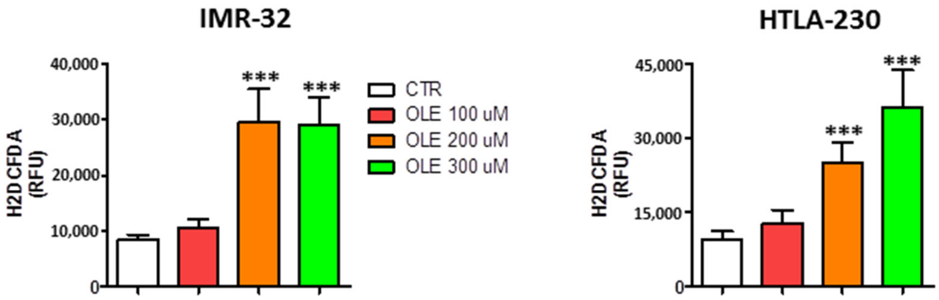

Supplementary Figure S7. Increased production of total ROS after OLE treatment. Data show the results of ROS production as assessed by collecting the fluorescence of H2DCFDA. Green fluorescence has been measured by the use of the TECAN micro-plate reader, Infinite 200. (485 nm/535 nm, excitation / emission); *** $p < 0.001$. RFU: relative fluorescence unit.

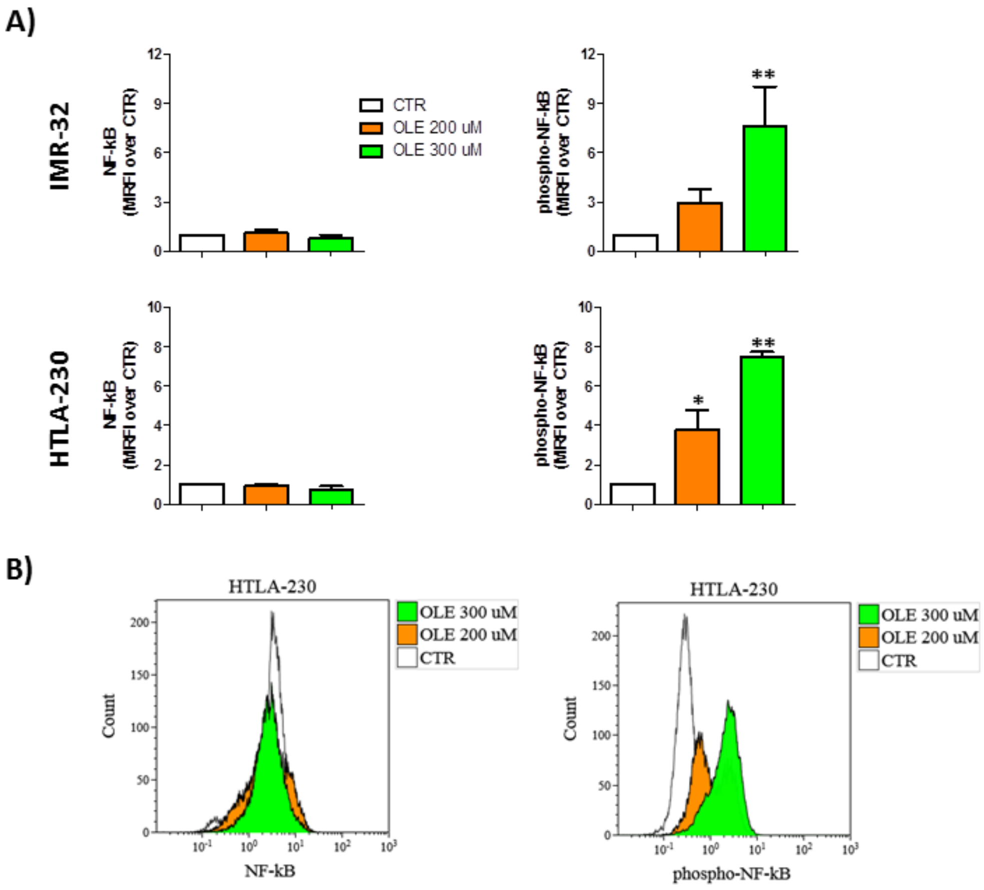

Supplementary Figure S8. Up-regulation of phospho-NF-kB after OLE treatment. (**A**) Data show the results of NF-kB and phospho-NF-kB protein expression levels determined by FCM as MRFI over CTR. (**B**) Representative FCM analyses of NF-kB and phospho-NF-kB protein expression levels. Data are expressed as mean ± SD (* $p < 0.05$, ** $p < 0.01$ vs CTR).

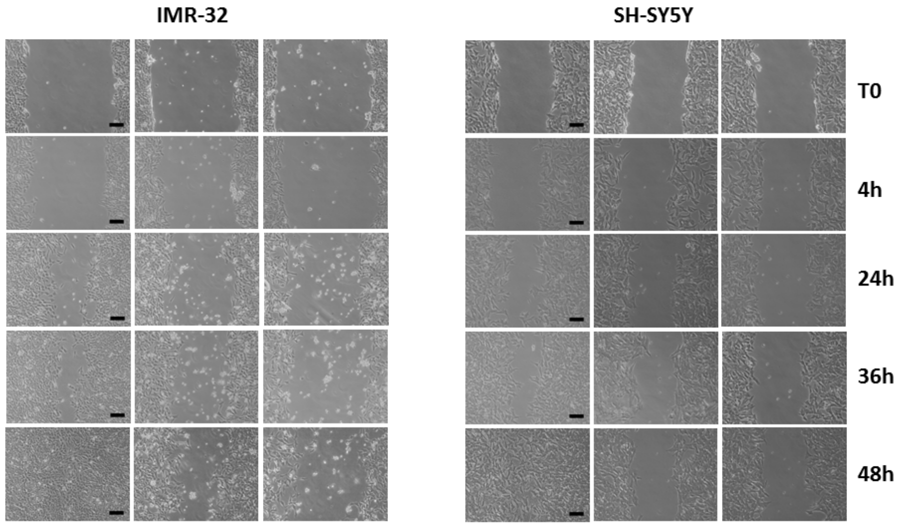

Supplementary Figure S9. Inhibition of migration of IMR-32 and SH-SY5Y cell lines after OLE treatment. Photographs show the progressive closure of the wound over time, in control untreated compared to OLE-treated cells. Scale bar: 100 µm.
